# The tonoplast – where sweetness is dispensable

**DOI:** 10.1093/jxb/erw074

**Published:** 2016-03-07

**Authors:** Richard Strasser

**Affiliations:** Department of Applied Genetics and Cell Biology, University of Natural Resources and Life Sciences, Muthgasse 18, 1190 Vienna, Austria

**Keywords:** Asparagine-linked oligosaccharides, glycoprotein, glycosylation, integral membrane protein, lysosome, membrane protein, tonoplast, vacuole

**The proteolytic machinery of certain cellular organelles is potentially harmful to resident proteins. In mammals, the large luminal domains of integral lysosomal proteins are heavily glycosylated to protect them from degradation. In this issue of *Journal of Experimental Botany* (pages 1769–1781) Pedrazzini and co-workers reveal that plants are very different.**

Glycosylation is one of the most common protein modifications in all eukaryotes. Resident proteins of different intracellular organelles including the endoplasmic reticulum (ER), Golgi apparatus and hydrolysing compartments are often glycosylated at multiple sites with oligosaccharide chains of varying length and composition. The attached carbohydrates modulate protein stability, activity and trafficking and mediate protein–protein interactions. *N*-glycosylation, the most prevalent form of protein glycosylation in eukaryotes, is defined by the covalent linkage of an oligosaccharide (*N*-glycan) to selected asparagine residues of newly synthesized secretory proteins as they are translocated into the ER. Once attached to the polypeptide chain, the *N*-glycans play crucial roles in protein folding and quality control processes (Helenius and Aebi, 2001) – for example, they regulate a specific cell death event in Arabidopsis (de Oliveira *et al.*, 2016).

Immediately after the transfer to the protein, *N*-glycans are subjected to step-wise processing reactions that alter the composition of the *N*-glycan and by doing so control the fate of glycoproteins. As a consequence, the exposure of a defined glyco-code can promote protein folding or alternatively serve as a signal for the degradation of terminally misfolded or orphan glycoproteins. In principle, these essential *N*-glycan-dependent processes look highly conserved between animals and plants (Hong *et al.*, 2012; Hüttner and Strasser, 2012). By contrast, in the Golgi apparatus where the *N*-glycans acquire their final composition (referred to as complex *N*-glycans) *N*-glycan processing steps differ substantially in eukaryotes. A large variety of structurally and functionally diverse complex oligosaccharides are generated in the Golgi in mammals. In different plant species, on the other hand, only a limited number of complex *N*-glycan structures have been reported so far (Wilson *et al.*, 2001). An outstanding question is therefore whether the reduced structural diversity of Golgi-processed *N*-glycans in plants also leads to fundamental differences in the biological role of these protein-linked carbohydrates.

While most of the machinery and pathways involved in biosynthesis and processing of *N*-glycan structures have been identified, the study of the biological role associated with distinct plant *N*-glycan structures on individual proteins has proved challenging so far (Strasser, 2014). More detailed and comprehensive (glyco)proteomic approaches of different organelles are needed for understanding the structure and function of glycoproteins in a specific cellular environment. Now Pedrazzini *et al.* (2016) reveal that the Arabidopsis tonoplast is almost completely lacking glycoproteins with complex *N*-glycans and even proteins with ER-derived *N*-glycans are highly underrepresented.

## Mammals and angiosperms

Existing data from proteomic studies of the Arabidopsis tonoplast and plasma membrane were subjected to bioinformatic analysis and compared to similar datasets from rat lysosomal and plasma membranes. A refined *in silico* analysis procedure was established to identify only the putative *N*-glycosylation sites that are accessible in the selected membrane proteins and face the lumen of the ER where they can be subjected to glycosylation. The comparison of datasets revealed a clear preference for the presence of these accessible *N*-glycosylation sites in Arabidopsis plasma membrane proteins. Such variation was not obviously present in the mammalian proteome samples analysed, highlighting clear differences between the kingdoms.

**Fig 1.**
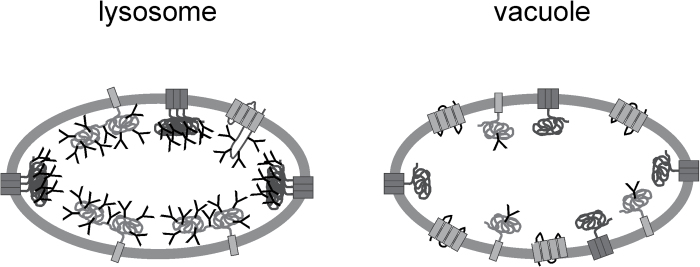
A dense glycoprotein coat has been observed in lysosomes – this protects integral membrane proteins from degradation. By contrast, the tonoplast in Arabidopsis leaves appears depleted from large amounts of glycoproteins with multiple *N*-linked glycans. ‘Y’ indicates the presence of glycans on membrane proteins.

In agreement with the *in silico* data, cell fractionation and immunofluorescence microscopy demonstrated the near complete absence of glycoproteins in the Arabidopsis tonoplast. While the scarcity of glycoproteins with complex *N*-glycans could be the consequence of a plant-specific protein trafficking route between the ER and the hydrolytic organelles that bypasses the Golgi (Pedrazzini *et al.*, 2013), this cannot explain the reduced number of glycoproteins with ER-type glycosylation in the tonoplast (see Fig. 1). In line with their findings, soluble vacuolar glycoproteins with Golgi-modified *N*-glycans are well known, but the majority of characterized heavily glycosylated membrane proteins are receptors found at the plasma membrane in plants (Häweker *et al.*, 2010; Hong *et al.*, 2012).

## Bad news for plant glycobiologists

In lysosomes, several of the most abundant membrane proteins, such as lysosome-associated membrane proteins (e.g. LAMP-1), are heavily glycosylated. A previous study demonstrated that a deglycosylated LAMP-1 variant is less stable *in vivo* in mammalian cells than the glycosylated one (Kundra and Kornfeld, 1999), underscoring the importance of *N*-glycosylation for protection from proteolytic degradation. Moreover, the heavily glycosylated integral membrane proteins presumably also constitute part of the thin glycocalyx that lines the inner membrane of lysosomes (Neiss, 1984; Wilke *et al.*, 2012). Although direct experimental evidence is missing, this carbohydrate coat has been postulated to protect the limited lysosomal membrane from degradation by the proteases active in the lysosome lumen. In addition, the glycans from the integral membrane proteins may serve as a barrier that has to be overcome to mediate specific transport processes like the export of cholesterol from the lysosomes (Li *et al.*, 2015).

LAMP-1 and other lysosomal membrane proteins are not only *N*-glycosylated, but also contain several *O*-linked glycans which turn out to play a similar role for the regulated release of content (Wilke *et al.*, 2012; Li *et al.*, 2015). Despite the fact that only *N*-glycosylation was investigated in the study from Pedrazzini and colleagues (2016), it is unlikely that other types of protein glycosylation safeguard tonoplast proteins from degradation by vacuolar hydrolases. *O*-glycosylation, the second major type of protein glycosylation, is fundamentally different between mammals and plants and the whole machinery for mucin-type mammalian *O*-glycosylation is not present in plants (Strasser, 2012). Plant-specific modifications of *O*-glycosylation sites have been described, but the attached glycan moieties are mainly present on cell wall proteins and typically not found on vacuolar proteins (Tan *et al.*, 2012). Consequently, it seems that a similar *N*- or *O*-glycan shield does not contribute to the protection of tonoplast proteins from intracellular proteolysis in plants.

So how are the integral proteins protected from proteolytic degradation in the vacuole? A clue to answer this question may arise from the observation by Pedrazzini *et al.* (2016) that long luminal polypeptide domains are not very frequent in the known tonoplast proteome. Evolutionary constraints may therefore have favoured the formation of another glycan-independent mechanism to protect proteins from harmful intracellular activities in plants. From the perspective of a plant glycobiologist this finding is not fruitful, but sometimes we just have to accept that proteins do well in the absence of sugars.
